# Epstein–Barr Virus Driven Hodgkin's Lymphoma after a Short Course of Daratumumab Treatment for Relapsed Multiple Myeloma

**DOI:** 10.1155/2023/6669174

**Published:** 2023-12-18

**Authors:** Moeen Mohammadi-Oroujeh, Ansa Mehreen, David L. Grinblatt

**Affiliations:** ^1^Department of Internal Medicine, University of Chicago (NorthShore), Chicago, IL, USA; ^2^Department of Pathology, University of Chicago (NorthShore), Chicago, IL, USA; ^3^Division of Hematology, Department of Medicine, NorthShore University HealthSystem, Evanston, IL, USA

## Abstract

In this case, we describe the potential risk of developing an infectious complication leading to a secondary malignancy after a short course of immunotherapy. We report a patient who presented with Epstein–Barr virus (EBV) driven Hodgkin's lymphoma after treatment with a short course of daratumumab along with pomalidomide and dexamethasone for relapsed multiple myeloma. Although there have been limited documented cases of daratumumab treatment leading to EBV reactivation, in patients presenting with infectious symptoms or neutropenia on a daratumumab-based regimen, testing for EBV should not be overlooked.

## 1. Introduction

Daratumumab, an anti-CD38 IgG antibody, was first approved for the treatment of multiple myeloma (MM) in 2015 [1]. In patients pretreated with lenalidomide who developed relapsed or refractory MM, the regimen of daratumumab along with pomalidomide and dexamethasone (DPd) has been shown to be an effective treatment option [2].

In this report, we present a patient who received treatment for relapsed MM with DPd and developed Epstein–Barr virus (EBV) driven Hodgkin's lymphoma (HL) after only two weeks of therapy.

## 2. Case Summary

The patient we describe was diagnosed with active myeloma at age 72 after developing right-sided rib pain which prompted a skeletal survey that showed lytic lesions in the skull, ribs, and femur. He was subsequently treated with 3 rounds of bortezomib, lenalidomide, and dexamethasone. His repeated bone marrow biopsy revealed 25% plasma cells; therefore, the regimen was changed to carfilzomib, lenalidomide, and dexamethasone for 3 cycles. This led to a complete response demonstrated by the resolution of his paraprotein and a reduction of bone marrow plasma cells to 4.8%. He then received an autologous stem cell transplant after high-dose melphalan treatment. The patient remained in a complete response on lenalidomide maintenance therapy for 6 years.

At age 79, on routine serum protein electrophoresis, he was found to have two new IgG kappa paraproteins with concentrations of 0.3 g/dl and 0.6 g/dl. This prompted a bone marrow biopsy which revealed 17% plasma cells, some of which were atypical in appearance. Treatment with DPd was started with daratumumab 8 mg/kg weekly and dexamethasone along with 14 days of pomalidomide.

However, soon after initiation of therapy, treatment was held due to palpitations, tachycardia, and syncope secondary to atrial fibrillation followed by neutropenia. On week five, our patient was admitted for neutropenic fever with an unrevealing infectious workup and was discharged after receiving filgrastim but was readmitted the following week for a mechanical fall.

**Figure 1:**
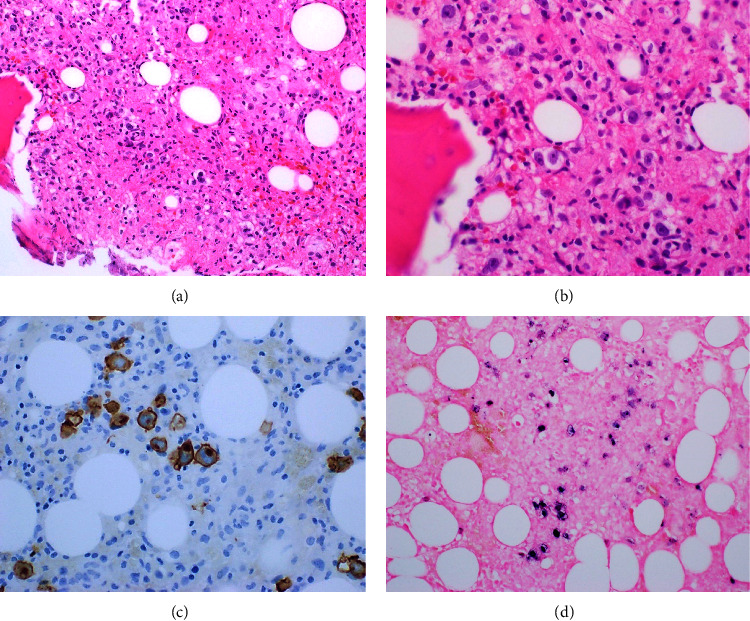
(a, b) Cellular bone marrow with scattered large mononuclear and binuclear cells in an inflammatory background on H & E. (c) CD30 highlighting the Hodgkin's cells. (d) EBER-ISH positive cells (blue chromogen).

**Figure 2:**
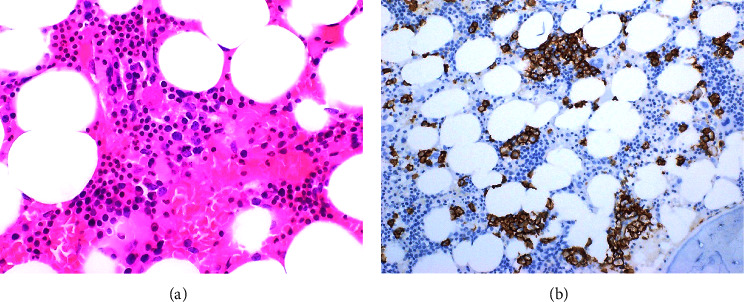
(a) Clustering of plasma cells in bone marrow on H & E. (b) CD138 highlighting the plasma cell clusters.

Due to the recurrence of fevers, a repeat bone marrow biopsy was performed (8 weeks after the pretreatment marrow) which revealed a mildly hypercellular marrow with cellularity comprised primarily of a diffuse to focally nodular histiocytic/granulomatous proliferation. Admixed within this proliferation were scattered intermediate-large mononucleate to focally binucleate cells with ovoid nuclear contours, intermediate chromatin pattern, and prominent centrally located nucleoli. The large cells were positive for CD15 and CD30 in a membranous and Golgi pattern and Epstein–Barr encoding region (EBER) on special stains (Figure 1). This was consistent with HL driven by EBV. The prior bone marrow biopsy did not reveal any significant HL-related changes (Figure 2). Further testing showed an EBV viral load of 80500 IU/ml by PCR, and serologies were positive for IgG and negative for IgM against EBV indicating reactivation. Subsequent paraprotein analysis and bone marrow testing showed no evidence of MM warranting additional treatment.

The newly diagnosed HL and EBV were treated with a dose of brentuximab that led to the resolution of fevers and an undetectable EBV viral load one month later. He continued to have improvement of the HL with brentuximab infusions. However, one year later at the age of 80, he was readmitted for a fall and pancytopenia after worsening of his HL led to an escalation of treatment and subsequently died due to septic shock.

## 3. Discussion

EBV commonly causes mononucleosis-type symptoms and has been shown to lay dormant in memory B-cells after acute infection for the life of the host. A diverse group of malignancies including Burkitt's, Hodgkin's, and diffuse large B-cell lymphoma as well as posttransplant lymphoproliferative disorders has been associated with EBV. Based on clinical and *in vitro* data, EBV-driven HL is thought to be caused by a dysregulation of B-cells in germinal centers. This lymphocytic proliferation is likely due to a lack of functional B-cell receptors and the incorporation of latent membrane proteins that prevent apoptosis [3].

In addition, immunosuppression appears to be implicated in EBV-driven lymphomas as is evident by an increased incidence in the elderly as well as posttransplant lymphoproliferative disorders, where patients are on medications that impede the immune system. In both populations, immunosuppression impairs the ability to control latent EBV leading to the reactivation and development of B-cell tumors [3].

Not many cases of EBV-driven lymphoma in the setting of daratumumab treatment have been documented. Two available published cases were after treatment with daratumumab along with lenalidomide and dexamethasone (DRd). One of these cases was reported in a patient from the GEN503 trial (phase 1, 2 approval trials for DRd) [4]. In this instance, the patient was treated with 8 cycles of DRd and was found to be in complete response; however, 40 months later while still on the DRd regimen, they were diagnosed with EBV-driven lymphoma based on slightly elevated EBV PCR levels [5]. An additional case was reported in a subgroup analysis of the POLLUX trial (phase 3 approval trials for DRd) focusing on East Asian patients, where the authors described a patient who developed an EBV-driven lymphoproliferative disorder; however, the type of lymphoma and treatment time was not published [6, 7].

Both cases of EBV-driven lymphoma after therapy with daratumumab were in combination with lenalidomide and dexamethasone. There are no documented reports of patients treated with a short course of DPd developing EBV-driven HL as described in our patient. There are data showing that thalidomide analogs may lead to EBV reactivation *in vitro*; however, no clinical cases have been described and our patient had been on lenalidomide for over 6 years [8]. Another possible explanation could be the dexamethasone in the regimen [9], although this is unlikely as the patient was on similar glucocorticoid doses prior without issue.

## 4. Conclusion

In conclusion, we describe the potential risk of developing an infectious complication leading to a secondary malignancy after a short course of daratumumab along with pomalidomide and dexamethasone. The incidence of EBV reactivation in patients on treatment with daratumumab is yet to be studied. However, in patients presenting with infectious symptoms or neutropenia on a daratumumab-based regimen, testing for EBV should not be overlooked.
